# Global research trends and insights in acupuncture randomized controlled trials: a bibliometric analysis

**DOI:** 10.3389/fmed.2026.1762387

**Published:** 2026-03-24

**Authors:** Menglong Zhang, Weiwei Zhang, Hongbo Jia, Songjiao Li, Kangchen Lei, Mengqi Li, Shuwen Shi, Yutong Dong, Zitong Li, Yuying Wang, Xibin Xu, Wenjun Zhao, Wei Liu, Sha Yang, Jian Liu, Li Li, Xiaonong Fan

**Affiliations:** 1 First Teaching Hospital of Tianjin University of Traditional Chinese Medicine, Tianjin, China; 2 National Clinical Research Center for Chinese Medicine, Tianjin, China; 3 Tianjin Key Laboratory of Acupuncture and Moxibustion, Tianjin, China; 4 Tianjin Institute of Acupuncture and Moxibustion, Tianjin, China; 5 Laboratory of Dose-Effect Relationship, National Administration of Traditional Chinese Medicine, Tianjin, China

**Keywords:** acupuncture, bibliometric analysis, global trends, randomized controlled trial, RCTs

## Abstract

**Objective:**

To identify the research status, major contributors, and emerging frontiers in acupuncture randomized controlled trials (RCTs) through bibliometric analysis, assisting clinicians, researchers, and policymakers in rapidly capturing valuable research hotspots and potential directions.

**Methods:**

Articles related to acupuncture RCTs published between January 1, 2010, and December 31, 2024, were retrieved 1,908 articles from the Web of Science Core Collection and Scopus. Multiple software tools (Origin 2021, CiteSpace, and VOSviewer) have been integrated to comprehensively visualize relationships among authors, journals, keywords, institutions, and countries.

**Results:**

The number of acupuncture RCTs grew steadily from 59 articles in 2010 to a peak of 205 in 2022, later stabilizing at 160. China led in output (1,096 articles), followed by the United States (263) and South Korea (216). Leading institutions included Beijing University of Chinese Medicine (166 articles; 2,084 citations) and Kyung Hee University (112 articles; top non-Chinese institution). Chinese scholars Liu Zhishun (most publications) and Liu Cunzhi (most citations) were the most prolific and highly cited researchers, respectively. The keywords of greatest interest were “Acupuncture” (1,188 times) and “Randomized Controlled Trial” (439 times). The next three most frequent keywords were “Electroacupuncture” (409 times), “Management” (291 times), and “Pain” (277 times). Burst keywords were “guidelines,” “sleep,” and “diagnosis” (2021–2022). *Trials* (274 articles) and *Acupuncture in Medicine* (H-index 26/2,099 citations) were the primary publishing journals.

**Conclusion:**

This study conducted a multidimensional analysis of the current state of acupuncture RCT research, revealing key research domains, hotspots, and potential trends. Building on these findings, we propose the following directions for future investigations: (1) identifying potential conditions for acupuncture through four classification approaches: conditions with conflicting research findings, conditions for which a body of evidence is emerging but has not yet reached a broad consensus, conditions for which modern medicine currently has no superior treatment options, and conditions where acupuncture can further expand its application; (2) key factors influencing acupuncture efficacy, such as acupoint prescription, core acupuncture parameters and the dose-effect relationship should be explored; (3) research quality and reliability should be ensured by adhering to rigorous methodological design and reporting standards, including appropriate selection of control groups and outcome measures.

## 1 Introduction

Acupuncture is currently widely used in multiple countries (1). To further verify its efficacy and safety, research on acupuncture is growing rapidly. Currently, randomized controlled trials (RCTs) aim to provide high-level evidence for clinically relevant questions. It can be used to validate the efficacy and safety of acupuncture effectively (2). Over the past few decades of acupuncture development, the number of RCTs on acupuncture has increased rapidly (3). Acupuncture has also been validated through RCTs to have moderate or large effects on eight diseases or conditions (1). Thus, the analysis of acupuncture RCTs facilitates the clinical translation of acupuncture for dominant diseases and enables the exploration of potential acupuncture dominant diseases.

Despite the increasing number of publications on acupuncture RCTs, high-level and high-quality evidence remains scarce (4). Most studies have primarily focused on verifying the efficacy of acupuncture. Many potential scientific questions remain unresolved (core factors in acupuncture efficacy, methodological issues, etc) (5). However, identifying novel research topics represents the first step toward generating high-quality evidence. Therefore, identifying innovative research directions and enhancing study quality to access more scientifically rigorous evidence-based findings remain urgent priorities (6).

Bibliometric analysis is the cross-cutting science of quantitatively analyzing all knowledge carriers via mathematical and statistical methods. A large amount of literature in the research area can be visualized and analyzed using this method (7). By analyzing the co-occurrence of countries, authors, keywords, etc., it can help researchers quickly identify hotspots and trends in current research and explore potential scientific issues (8). It translates a vast body of literature into clear, evidence-based insights, thereby enabling the design of more targeted, methodologically sound, and impactful RCTs. This, in turn, is fundamental for generating the robust scientific evidence required to solidify the role of acupuncture in global healthcare.

CiteSpace and VOSviewer are currently the most commonly used bibliometric analysis software (9, 10). CiteSpace is a JAVA application for data analysis, literature visualization, and mutation analysis. The core functions of this software are burst detection, betweenness centrality, and heterogeneous networks (11, 12). It can be used to visualize the research field and dig into the hot spots of the field development through clustering and mutation analysis. VOSviewer is a free bibliometric analysis software created by Nees Jan van Eck and Ludo Waltman (Leiden University) to construct and analyze scientific knowledge graphs (13). This software can build co-occurrence maps of authors, keywords, and institutions using VOSviewer mapping technology, which is more intuitive when analyzing at least 100 documents (7). VOSviewer can also be used to build and visualize co-occurrence networks of essential terms through its text-mining capabilities (14).

This study employs bibliometric software (VOSviewer and CiteSpace) to analyze publications indexed in the Web of Science Core Collection (WOSCC) and Scopus between 2010 and 2024. By analyzing annual publication trends, journals, research areas, keywords, authors, affiliations, and countries, this analysis aims to delineate the current research landscape, emerging hotspots, and evolving trends in RCTs of acupuncture. This study aims to provide novice researchers entering the field of acupuncture RCTs with a comprehensive knowledge map, enabling them to systematically grasp the current research landscape. Simultaneously, it offers recommendations to experienced researchers to promote the production of higher-quality RCTs.

## 2 Methods

### 2.1 Data sources and search strategies

This study adhered to the standards of bibliometric research, employing only systematic methods for database retrieval, study selection, and data extraction. However, it did not conduct a comprehensive analysis of clinical outcomes, perform risk of bias (RoB) assessments for individual trials, or formally evaluate the methodological quality of randomized controlled trials.

We conducted a systematic search of the WOSCC and Scopus databases on August 20, 2025, to identify acupuncture randomized controlled trials (RCTs) published between January 1, 2010, and December 31, 2024.

For WOSCC, the following search strategy was applied: TS = ((acupuncture OR acupuncture therapy OR acupuncture points OR manual acupuncture OR auricular acupuncture OR scalp acupuncture OR electroacupuncture OR acupuncture treatment OR pharmacoacupuncture treatment OR acupotomy) AND (randomized controlled trial OR RCT OR random allocation)). The search was refined by document type (Articles only) and language (English). For Scopus, the search strategy was adapted as follows: TITLE-ABS-KEY (“acupuncture” OR “acupuncture therapy” OR “acupuncture points” OR “manual acupuncture” OR “auricular acupuncture” OR “scalp acupuncture” OR “electroacupuncture” OR “acupuncture treatment” OR “pharmacoacupuncture treatment” OR “acupotomy”) AND TITLE-ABS-KEY (“randomized controlled trial” OR “RCT” OR “random allocation”). The results were limited to articles (DOCTYPE ar) published in English. The detailed search strategies are presented in Supplementary Table S1.

### 2.2 Data processing and cleaning

All records retrieved from WOSCC and Scopus were imported into R 4.5.0 for merging, deduplication, and cleaning. The R packages used included *tidyverse* (for data manipulation), *stringdist* (for fuzzy matching), and *stringr* (for string processing).

#### 2.2.1 Merging and deduplication

Records from the two databases were combined using the *dplyr* package. Duplicate records were identified in a hierarchical manner:

*DOI-based matching*: Records with identical DOIs after case-insensitive trimming were considered duplicates. When a duplicate pair consisted of one record from WOSCC and one from Scopus, the WOSCC record was retained because of its more complete citation data; otherwise, the record with the richer metadata (e.g., more complete author keywords) was kept.

*Title-based fuzzy matching*: For records without a DOI (or with empty DOI fields), we performed fuzzy matching using the Levenshtein distance (calculated with the *stringdist* package). Titles were first converted to lowercase, stripped of punctuation, and trimmed of extra whitespace. Pairs with a similarity score > 0.9 and the same publication year were manually inspected; if they clearly referred to the same study, one record was removed. No records were excluded solely because of a missing DOI.

#### 2.2.2 Author name disambiguation

To ensure accurate author-level analysis, all author names were standardized to a consistent format (“Last Name, First Name”). For authors with multiple name variants (e.g., “Liu, Zhishun” and “Liu Zhi-shun”), the variant with the highest occurrence frequency was adopted as the standard name using custom string-processing functions in R. The complete list of merged name variants is provided in Supplementary Table S2.

#### 2.2.3 Institution name harmonization

Organizational Affiliations were extracted from address fields and cleaned to remove extraneous information (e.g., postal codes and department names). The cleaned names were then unified according to a manually curated synonym list. For example, “Kyung Hee Univ” and “Kyung Hee University” were merged into “Kyung Hee University.” The complete table is provided in Supplementary Table S3.

#### 2.2.4 Keyword standardization

Keywords were standardized using a predefined thesaurus to merge synonyms and spelling variants. For example “Metaanalysis” was merged into “Meta-analysis” and “Electro-Acupuncture” was merged as “Electroacupuncture.” The complete list is provided in Supplementary Table S4.

#### 2.2.5 Country/region name harmonization

To enable consistent country-level analysis, we unified regional and national designations as follows: “England,” “Scotland,” “Wales,” and “Northern Ireland” were reclassified as the United Kingdom. Articles originating from “Taiwan” (as listed in the databases) were included under China to reflect the research’s geographical scope without making any political statement. See Supplementary Table S5 for a detailed merged list of countries/regions.

### 2.3 Inclusion and exclusion criteria and final inclusion

**Figure 1:**
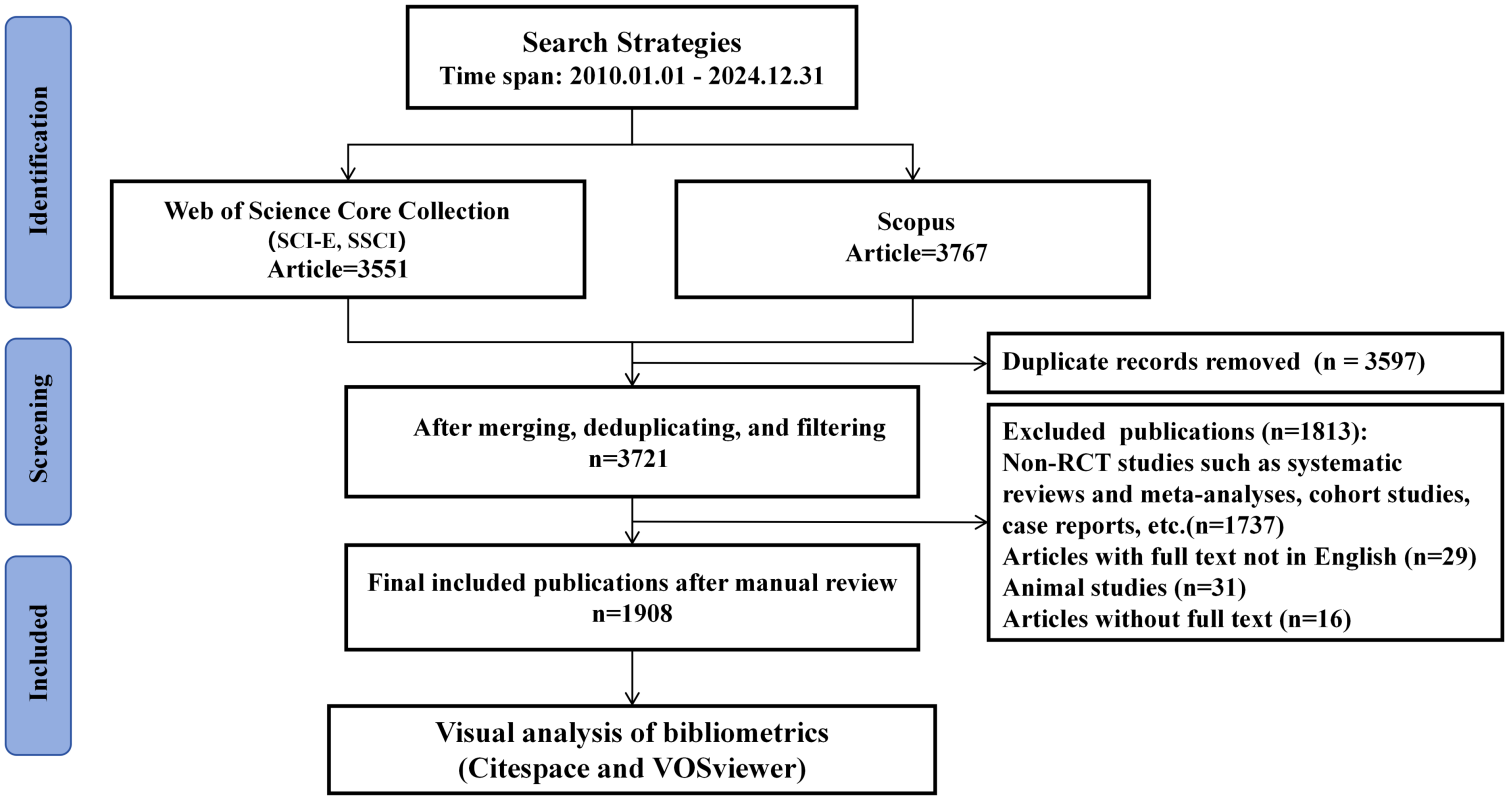
Research screening flowchart.

After two independent researchers (WWZ and HBJ) excluded animal studies, articles for which the full text was not available (lack of essential analytical information, such as incomplete research design or abstract details), articles with full text available not in English (including those with only the abstract in English), and non-RCT studies such as systematic evaluations and meta-analyses, cohort studies, and case reports, etc. It is worth noting that articles without full text were excluded because partial metadata (e.g., detailed author affiliations, research design) of these records were incomplete, which may lead to errors in subsequent bibliometric analysis (e.g., institution co-occurrence, keyword extraction). Due to the small number of such articles (*n* = 16), the potential selection bias caused by this exclusion was considered minimal. Any disagreements were resolved through discussion until a consensus was reached or until a third researcher was consulted. Finally, 1908 articles were included in this study (Figure 1).

### 2.4 Data analysis

We used Origin 2021 to intuitively analyze the annual number of publications. For the related table construction, we use Excel 2021. Moreover, we imported all filtered data into VOSviewer v.1.6.20 to visually analyze relationships among authors, journals, keywords, organizational affiliations, and countries. The corresponding results are generated using VOSviewer to perform co-occurrence, clustering, and visual analysis. Moreover, we used CiteSpace 6.4. R1 for keyword, organizational affiliation, and country analysis to obtain a more comprehensive view of research trends.

VOSviewer uses VOS mapping methods to reflect the visualization of similarity (10). We applied VOSviewer to construct the network map of bibliometric analysis that can be intuitively analyzed, in which the size of nodes represents the frequency of entries. The length of the connecting lines represents the correlation between the two entries. The thickness of the connecting lines indicates the strength of the link between nodes, and different colors indicate different clusters. The different clusters are automatically divided by the software according to the degree of association. The VOSviewer parameters are as follows: (1) The scale in visualization was set to 1.60. (2) The size variation in labels was set to 0.50, and the clustering graph was set to circles. (3) The size variation in the lines was set to 0.50, and the lines were presented as colored lines and straight lines. CitesSpace 6.4. R1 parameters are as follows: (1) time slice (2010 to 2024), year per slice. (2) In the selection of node type, we selected country, organizational affiliations, and burst keywords. (3) In terms of the strength of the link, we choose cosine, and in terms of the scope, we choose within slices. (4) We use the g-index as our selection criterion, and the factor k = 25. The detailed parameter settings for VOSviewer and CiteSpace are provided in Supplementary Table S6.

## 3 Results

### 3.1 Analysis of annual publications and trends

**Figure 2:**
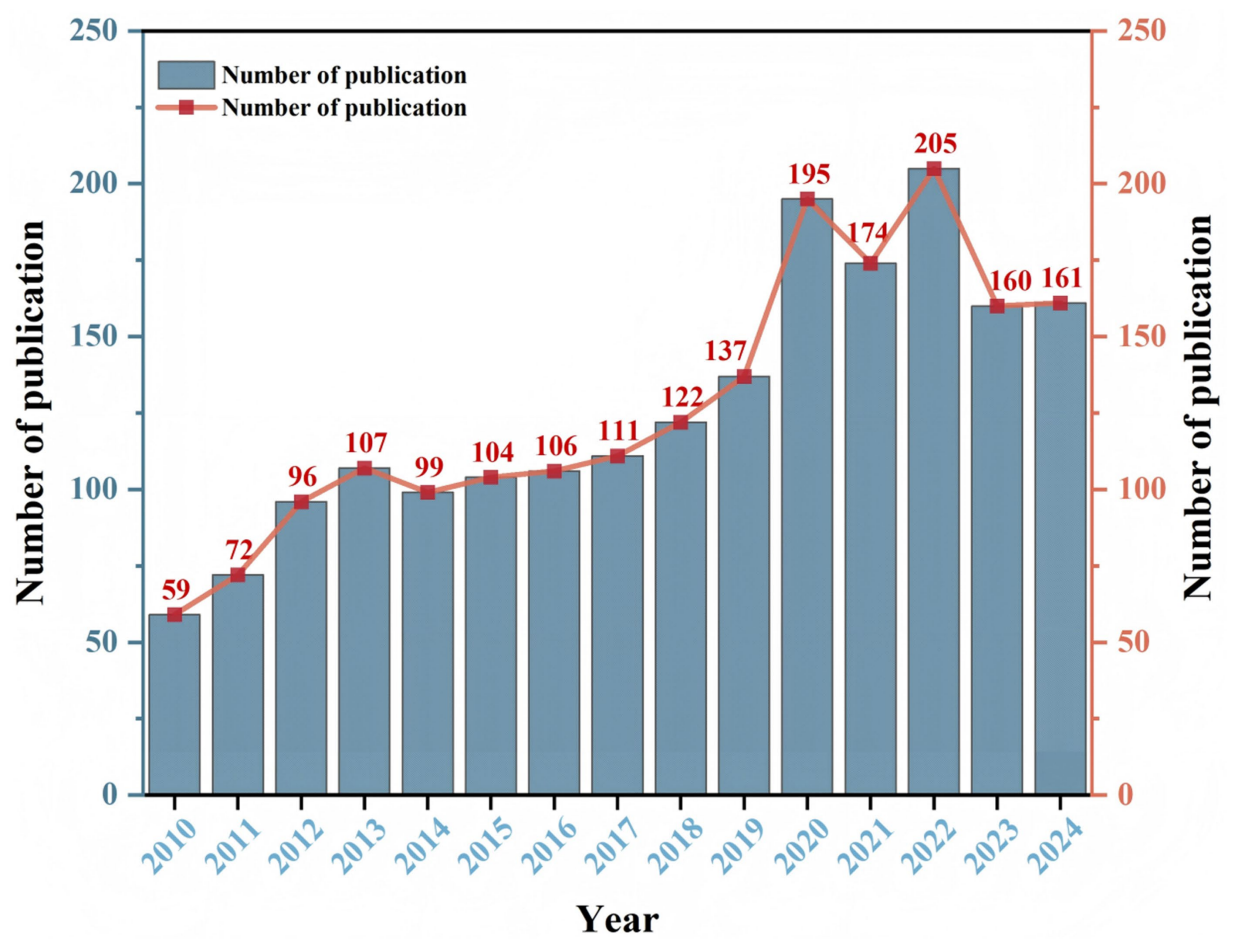
Trends in the number of publications of RCTs of acupuncture from 2010 to 2024.

As shown in Figure 2, from January 1, 2010, to December 31, 2024, a total of 1908 articles on randomized controlled trials of acupuncture were published. The number of relevant articles published in 2010, 2011, 2012, and 2014 was fewer than 100, and it increased to more than 100 after 2015, with yearly increases. Since the beginning of 2019, the number of articles has increased significantly, reaching a maximum of 205 articles in 2022. The number of publications has stabilized at approximately 160 in the last 2 years. Although the number of publications has fluctuated, the annual number of publications has increased.

### 3.2 Analysis of countries

**Figure 3:**
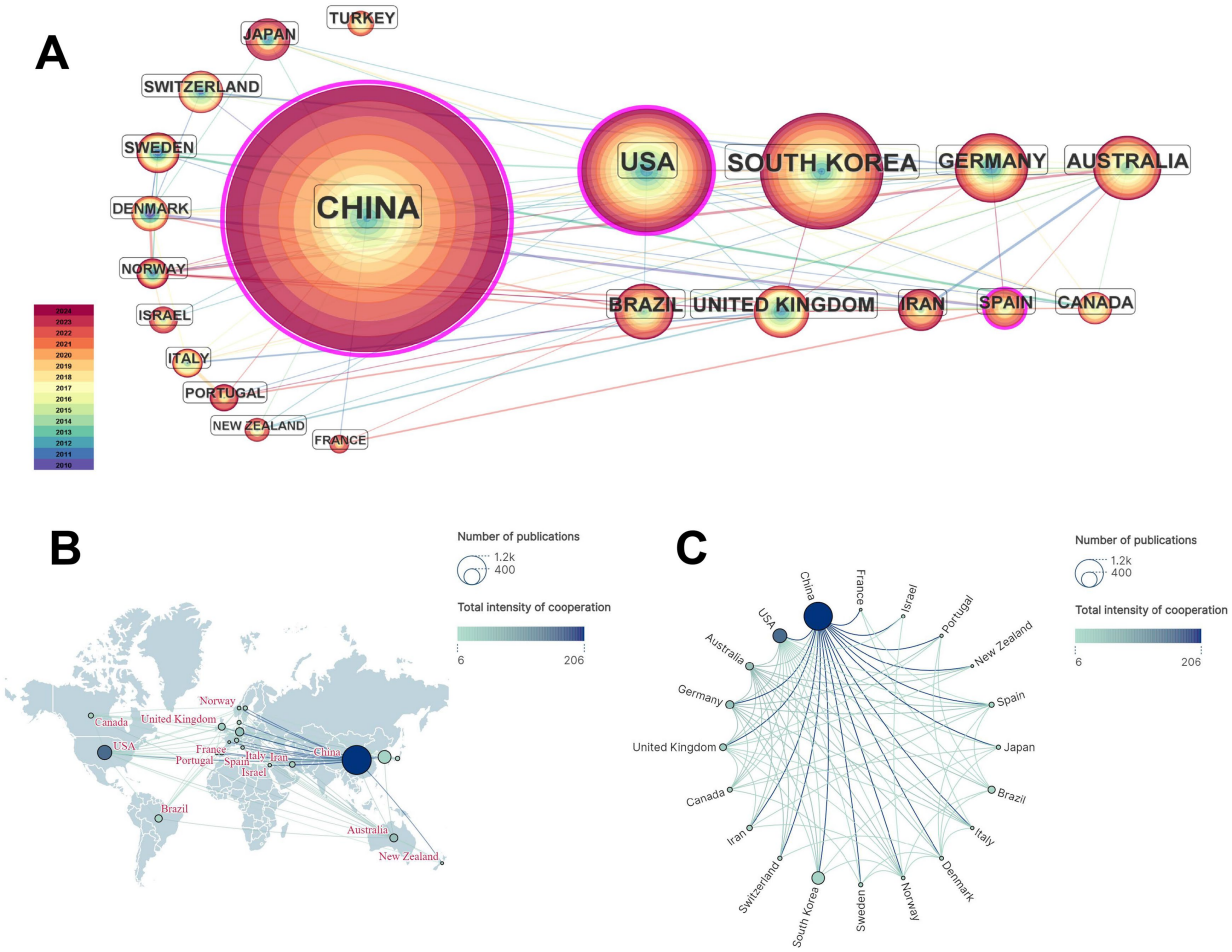
Network map of countries. **(A)** Country with more than 10 publications. **(B)** Geographic network map. **(C)** Country collaboration network map. Clustering methods: Spectral clustering. Threshold: Only countries appearing ≥10 times were included (*n* = 21). This cutoff was selected to focus on core research themes while maintaining network readability by excluding low-frequency terms. Node: The number of publications issued by countries; the larger the node, the greater the number of publications. The purple outer ring of a node indicates its centrality; a darker shade corresponds to greater centrality. Edge: The strength of collaboration between countries; the thicker the line, the stronger the collaboration.

To avoid data bias due to nonharmonization across countries, we harmonize Wales, Northern Ireland, Scotland, and England as the United Kingdom, and China, including Taiwan. Finally, a total of 46 countries issued publications, of which 21 countries issued more than 10 articles (Figure 3A; Supplementary Table S7). Among them, China, the USA, South Korea, Germany, and Australia are the top 5 countries in terms of the number of articles published (Table 1). As shown in Table 1, “Betweenness Centrality” refers to betweenness centrality calculated by CiteSpace. It measures the extent to which a country acts as a bridging node in the collaboration network. China has the highest number of publications (1,096) and the highest betweenness centrality (0.64). A value≥ 0.1 for betweenness centrality is usually considered indicative of greater influence within the network. Therefore, China is the country with the higher research productivity.

**Table 1:** Top 10 countries in terms of the number of publications.

Rank	Country	Number of publications	Betweenness centrality
1	China	1,096	0.64
2	USA	263	0.30
3	South Korea	216	0.02
4	Germany	90	0.05
5	Australia	83	0.06
6	Brazil	70	0.09
7	United Kingdom	63	0.06
8	Iran	41	0.08
9	Spain	36	0.11
10	Canada	34	0.05

As demonstrated in Figures 3B,C, the top five countries by publication count have stronger connections that radiate outward to other countries, indicating that acupuncture research has gained worldwide importance. Therefore, there is a need to strengthen links between countries to facilitate the production of high-quality evidence on acupuncture.

### 3.3 Analysis of organizational affiliations

**Figure 4:**
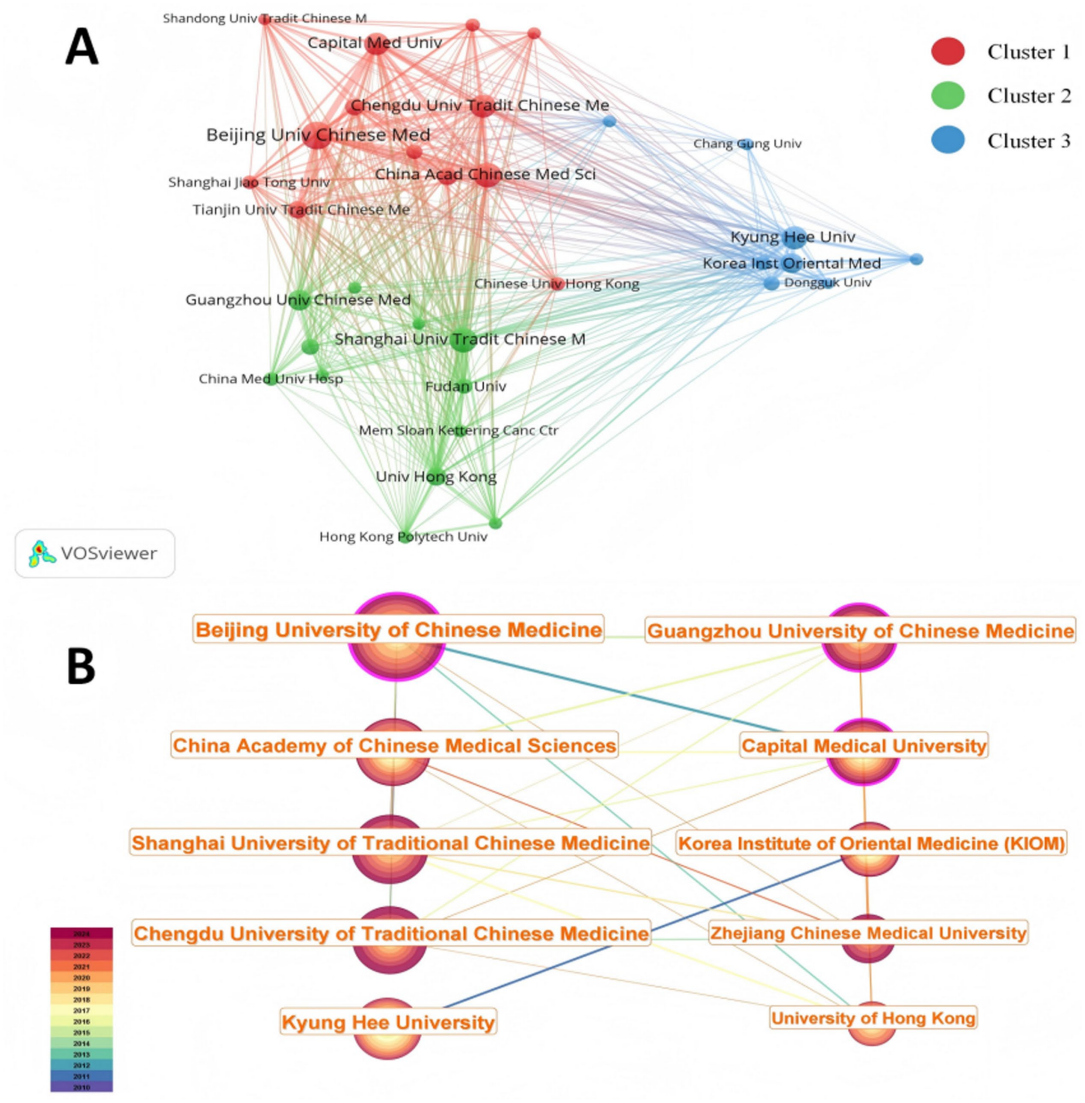
Network map of organizations. **(A)** Co-occurrence mapping of organizations with more than 20 publications based on citations, divided into three clusters. Aiming to identify high-output core organizations in the field of acupuncture RCTs. **(B)** Country collaboration network map. The top 10 organizations with the most publications are displayed to further illustrate the academic connections among core institutions. Clustering methods: VOSviewer (Modularity-based); CiteSpace (Spectral Clustering). Node: The number of publications issued by organizations; the larger the node, the greater the number of publications. The purple outer ring of a node indicates its centrality; a darker shade corresponds to greater centrality. Edge: The strength of collaboration between organizations; the thicker the line, the stronger the collaboration.

We used VOSviewer to analyze 2,110 affiliations, of which 33 have issued more than 20 publications (Figure 4A). The top 20 organizations with the highest number of publications and related information can be found in Supplementary Table S8. The top 10 organizations by publications are all from Asia, with eight in China and two in South Korea (Table 2). As shown in Table 2, “Betweenness Centrality” measures the extent to which an organization serves as a connecting node within cooperative networks. Beijing University of Chinese Medicine ranked first in publication output (166 articles) and exhibited the highest betweenness centrality (0.23). The China Academy of Chinese Medical Sciences followed with 121 publications and a betweenness centrality of 0.06 (Table 2). The number of citations represents, to some extent, the level of recognition of the publication. These two organizations also have the highest number of citations, 2084 and 1782, respectively (Table 2). Therefore, Beijing University of Chinese Medicine is an organization with higher research productivity and citation impact.

**Table 2:** The 10 organizations publishing the highest number of articles about RCTs of acupuncture.

Rank	Organization	Records	Citations	Betweenness centrality
1	Beijing University of Chinese Medicine	166	2084	0.23
2	China Academy of Chinese Medical Sciences	121	1782	0.06
3	Shanghai University of Traditional Chinese Medicine	121	1,522	0.05
4	Chengdu University of Traditional Chinese Medicine	116	1,276	0.08
5	Kyung Hee University	112	1,329	0.04
6	Guangzhou University of Chinese Medicine	110	561	0.15
7	Capital Medical University	96	1,465	0.12
8	Korea Institute of Oriental Medicine Kiom	76	979	0.04
9	Zhejiang Chinese Medical University	62	561	0.03
10	University of Hong Kong	60	1,249	0.05

The color of the line represents the ties between countries, with thicker lines representing stronger ties between Organizations. As shown in Figure 4A, organizations are grouped into three clusters based on geographic location, intensity of cooperation, number of citations, etc.: 13 organizations in cluster 1, mostly in northern China; 12 organizations in cluster 2, mostly in southern China; and cluster 3, mostly in Korea. The most representative organizations in each of the three clusters are Beijing University of Chinese Medicine, Shanghai University of Traditional Chinese Medicine, and Kyung Hee University.

In China, Beijing University of Chinese Medicine and China Academy of Chinese Medical Sciences are more closely linked. In Korea, Kyung Hee University (112 publications, 1,329 citations, and 0.04 betweenness centrality) and Korea Institute of Oriental Medicine Kiom (76 publications, 979 citations, and 0.04 betweenness centrality) are more closely connected (Figure 4B). Among them, Kyung Hee University has not yet developed links and cooperation with the top-ranked organizations in China. Accordingly, there is a need to enhance cooperation and linkages among the heads of organizations across countries to jointly promote the development of acupuncture.

### 3.4 Analysis of keywords

Keywords represent the research focus of the article and cluster analysis of them can help to reveal potential future research hotspots. We used VOSviewer to analyze the co-occurrence of 4,988 keywords and to modify and merge synonyms and improperly expressed words (e.g., Metaanalysis was replaced with Meta-analysis Electro-Acupuncture was replaced with Electroacupuncture). A total of 57 keywords with a frequency of occurrence of 40 or more were included in the analysis. The top 20 keywords with the highest frequency of occurrence are listed in Supplementary Table S9.

**Figure 5:**
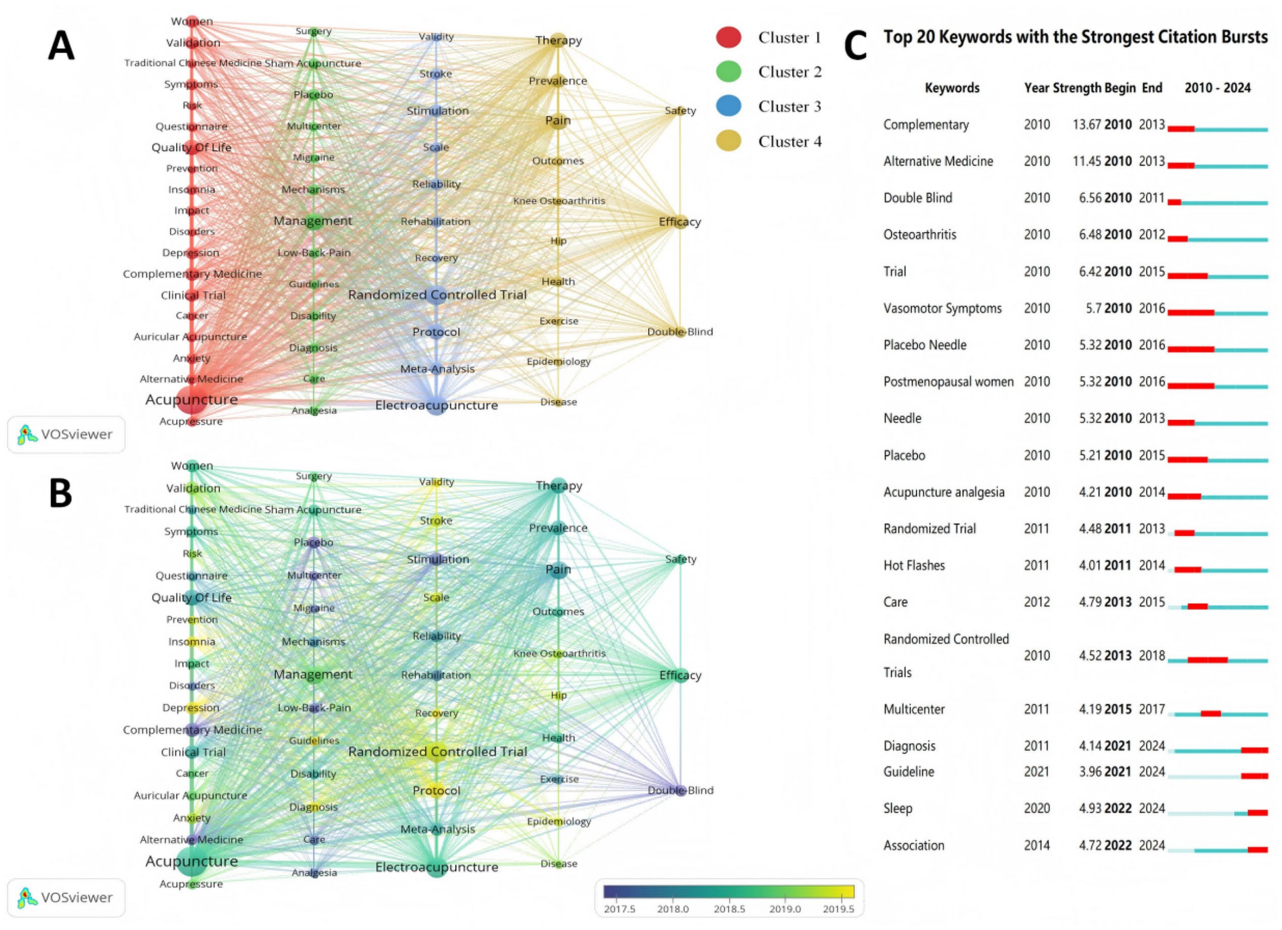
Graphical analysis of keywords. **(A)** Co-occurrence mapping of keywords that occur more than 40 times, divided into four clusters. To further refine the focus on core research themes and avoid an excessively dense visualization network, a threshold of 40 was chosen for keyword visualization. **(B)** Temporal view of keywords. **(C)** Keywords with the strongest citation bursts according to CiteSpace. Clustering Methods: VOSviewer (Modularity-based). Node: The frequency of keyword occurrences; a larger node indicates a higher occurrence frequency. Edge: The link strength between keywords; a thicker line indicates a closer relationship between keywords.

As illustrated in Figure 5A, the most concerned keywords are “Acupuncture” (1,188 times) and “Randomized Controlled Trial” (439 times). The next three most frequent keywords are “Electroacupuncture” (409 times), “Management” (291 times), and “Pain” (277 times). For a clearer identification of research trends, the keywords were categorized into 4 distinct clusters. Cluster 1 primarily reflects the application of acupuncture to improve the symptom burden and quality of life in specific patient populations, particularly those with chronic and psychosomatic diseases. Keywords such as “Women,” “Cancer,” “Depression,” “Anxiety,” and “Insomnia” indicate that research focuses on women’s health, adjunct cancer care, and mental health disorders. Meanwhile, “Auricular Acupuncture” and “Acupressure” demonstrate the diversity of acupuncture interventions. In terms of outcome measures, “Quality of Life” (195 times) and “Symptoms” highlight an emphasis on holistic improvements in patients’ subjective experiences and functional status. Cluster 2 places greater emphasis on methodological rigor and on translating evidence. “Sham Acupuncture” and “Placebo” represent the methodologically challenging elements in acupuncture RCT design, aimed at validating the specific efficacy of acupuncture. And “Multicenter,” “Guidelines,” and “Mechanisms” indicate that while generating high-quality evidence for inclusion in guidelines, it is also essential to explore their underlying mechanisms. Studies on “low back pain” and “migraine” often employ rigorous methodological designs, thereby yielding high-quality evidence of acupuncture efficacy. Cluster 3 centers on evaluating the efficacy of acupuncture in the rehabilitation of neurological disorders (particularly stroke) while emphasizing the standardization of assessment tools and research protocols. “Stroke,” “Rehabilitation,” “Recovery,” and “Electroacupuncture” (409 times) highlight the importance of electroacupuncture as a key intervention. And the terms including “Randomized Controlled Trial,” “Meta-analysis,” “Protocol,” “Validity,” “Reliability,” and “Scale” demonstrate the field’s emphasis on high-level study design, data synthesis, and the scientific rigor of evaluation methodologies. Cluster 4 focuses on validating the efficacy and safety of acupuncture in treating common musculoskeletal pain disorders, such as knee osteoarthritis, while also examining the disease burden from an epidemiological perspective. Keywords such as “Pain,” “Knee Osteoarthritis,” and “Hip” represent the most extensively studied pain conditions in this domain of acupuncture therapy. The research objective focuses on “Efficacy” and “Safety.” Keywords such as “Prevalence” and “Epidemiology” indicate the current research focus of this disease category.

Figure 5B visualizes the chronological distribution of keyword emergence frequencies, with node positioning reflecting the mean temporal coordinates in the research timeline. The chromatic intensity of nodes (yellow hue spectrum) correlates with keyword novelty, where heightened color saturation indicates greater temporal recency in the research focus. “Protocol” is the most cutting-edge keyword, appearing on average in 2020, suggesting that acupuncture randomized controlled trials are becoming more rigorous and that a standardized protocol is a prerequisite for conducting high-quality RCTs. The analysis of emergent words can reveal the dynamic evolutionary trend of a discipline and help to identify research hotspots. The burst detection analysis in Figure 5C reveals keyword emergence: a 2022 cluster comprising “Association” and “Sleep,” and a 2021 cluster featuring “Guideline” and “Diagnosis,” indicating current research frontiers in this field.

### 3.5 Analysis of journals and research areas

**Figure 6:**
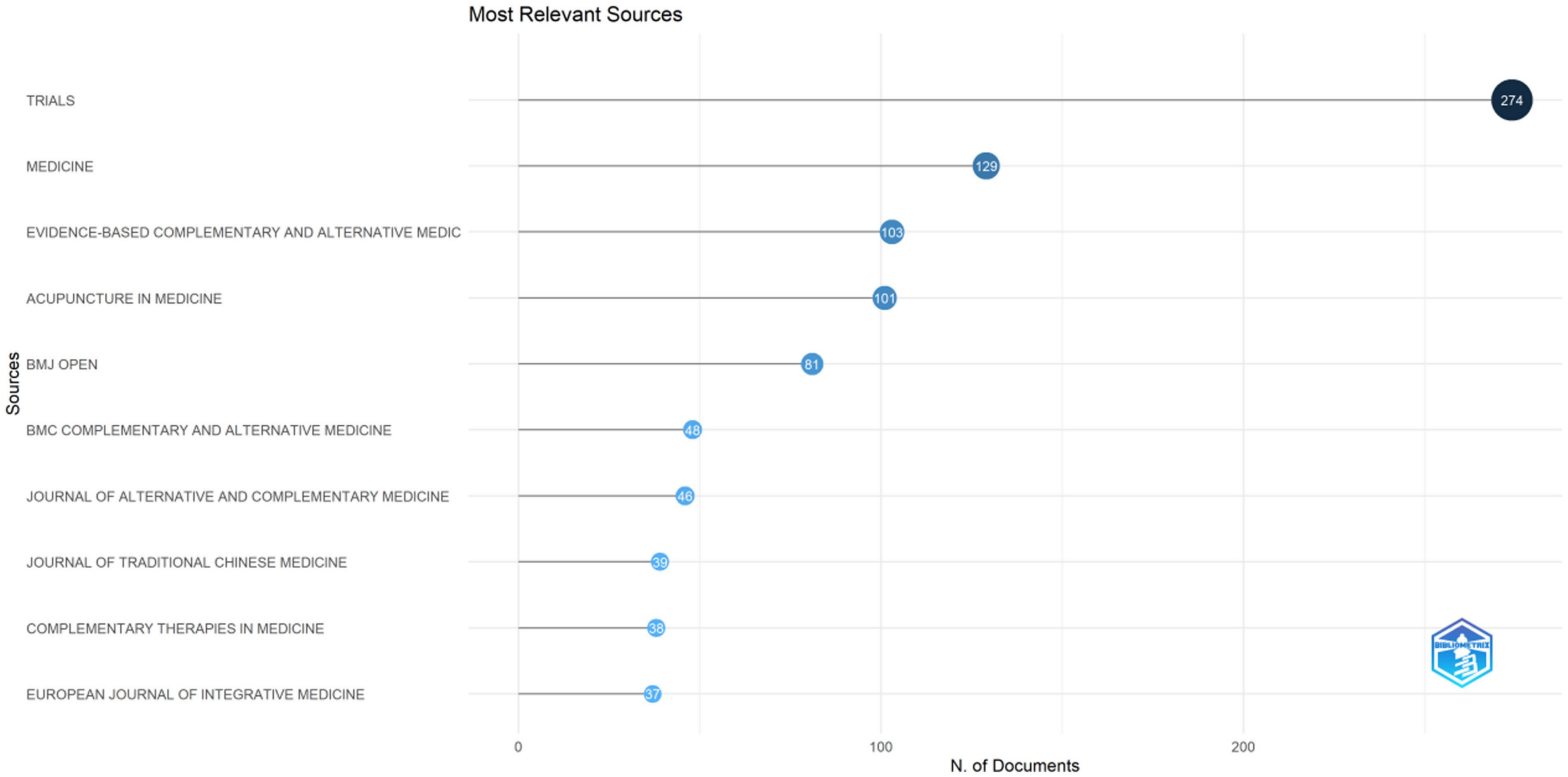
Top 10 journals in terms of publications. To further focus on the highly productive journals, a threshold of 10 was selected. The size of the node corresponds to the number of publications.

A total of 200 journals were searched for publications on RCTs of acupuncture. As shown in Figure 6 and Table 3, the top 10 journals, with a total of 897 publications, accounted for 47.01% of all articles published. The IF and H-index can be used to indicate a journal’s impact. The journal with the highest number of publications was *Trials* (IF = 2.0, H-index = 17), which published 274 publications with a TC of 2090. The journal with the highest H-index (15) and TC (2099) was *Acupuncture in Medicine* (IF = 2.6), with 101 publications. *BMC Complementary and Alternative Medicine* (TP = 48, TC = 934, H-index = 19) and *Complementary Therapies in Medicine* (TP = 39, TC = 583, H-index = 16) are the journals with the highest IF (3.5) (Table 3).

**Table 3:** Top 10 most productive journals about RCTs of acupuncture.

Rank	Journals	TP	TC	H-index	IF (2024)/JCR
1	Trials	274	2090	17	2.0/Q3
2	Medicine	129	666	13	1.4/Q2
3	Evidence Based Complementary and Alternative Medicine	103	1,255	19	0
4	Acupuncture in Medicine	101	2099	26	2.6/Q2
5	BMJ Open	81	385	10	2.3/Q2
6	BMC Complementary and Alternative Medicine	48	934	19	3.4/Q1
7	Journal of Alternative and Complementary Medicine	46	963	20	2.4/Q3
8	Complementary Therapies in Medicine	39	583	16	3.5/Q1
9	Journal of Traditional Chinese Medicine	39	373	14	2.2/Q2
10	European Journal of Integrative Medicine	37	187	8	1.7/Q3

TP, total number of publications; TC, total citations; IF, according to Journal Citation Reports (2024).

Research areas that can indicate the main research directions of acupuncture RCTs in recent years. Integrative Complementary Medicine and General Internal Medicine are the two areas with the highest numbers of articles, with 624 and 331, respectively. The disease areas designed are Neurology, Oncology, Anesthesiology, and Obstetrics and Gynecology. This result is similar to that of the keyword analysis (Table 4).

**Table 4:** The 10 most represented research areas in RCTs of acupuncture.

Rank	Research areas	TP
1	Integrative Complementary Medicine	624
2	General Internal Medicine	331
3	Research Experimental Medicine	314
4	Neurosciences Neurology	204
5	Oncology	80
6	Anesthesiology	59
7	Rehabilitation	52
8	Science Technology Other Topics	43
9	Obstetrics Gynecology	41
10	Health Care Sciences Services	39

Table 5 shows that the top ten most cited articles focused on four categories of diseases: (1) pain disorders (migraine (16), chronic knee osteoarthritis (17)); (2) oncotherapy-associated complications (joint pain related to aromatase inhibitors among women with early-stage breast cancer (18), cancer-related fatigue in patients with breast cancer (19), and vasodilatory symptoms in breast cancer (20)); (3) gynecological disorders (hyperandrogenism and oligomenorrhea/amenorrhea in women (21), polycystic ovary syndrome (22)); and (4) functional somatic syndromes (chronic and severe functional constipation and primary insomnia (23, 24)). In terms of intervention modalities. Interventional modalities were dominated by manual acupuncture (90%, *n* = 9), with electroacupuncture comprising the remaining 10% (*n* = 1). The most cited publication is Zhao L et al., published in *JAMA Internal Medicine* (IF = 22.5, Q1) on manual acupuncture intervention for migraine with 250 citations. Among them, 4 articles were published by Chinese authors, and the journal with the highest impact factor was *JAMA* (IF = 63.1, Q1).

**Table 5:** Top 10 most cited publications on RCTs of acupuncture.

First author	TC	Country	Journal (Year)	Intervention	IF (2024)/JCR
Zhao, Ling (16)	250	China	*JAMA Intern Med (2017)*	MA	23.3/Q1
Hershman, Dawn L (18)	212	USA	*JAMA (2018)*	MA	55/Q1
Crew, Katherine D (91)	202	USA	*J Clin Oncol (2010)*	MA	41.9/Q1
Liu, Zhishun (23)	200	China	*Ann Intern Med (2016)*	MA	15.2/Q1
Hinman, Rana S (17)	195	Australia	*JAMA (2014)*	MA	55/Q1
Molassiotis, A (19)	179	UK	*J Clin Oncol (2012)*	MA	41.9/Q1
Walker, Eleanor M (20)	155	USA	*J Clin Oncol (2010)*	MA	41.9/Q1
Yin, Xuan (24)	142	China	*Sleep Med (2017)*	MA	3.4/Q2
Jedel, Elizabeth (21)	140	Sweden	*Am J Physiol Endocrinol Metab (2011)*	EA	3.1/Q2
Wu, Xiao-Ke (22)	137	China	*JAMA (2017)*	MA	55/Q1

MA, manual acupuncture; EA, electroacupuncture.

### 3.6 Analysis of authors

**Figure 7:**
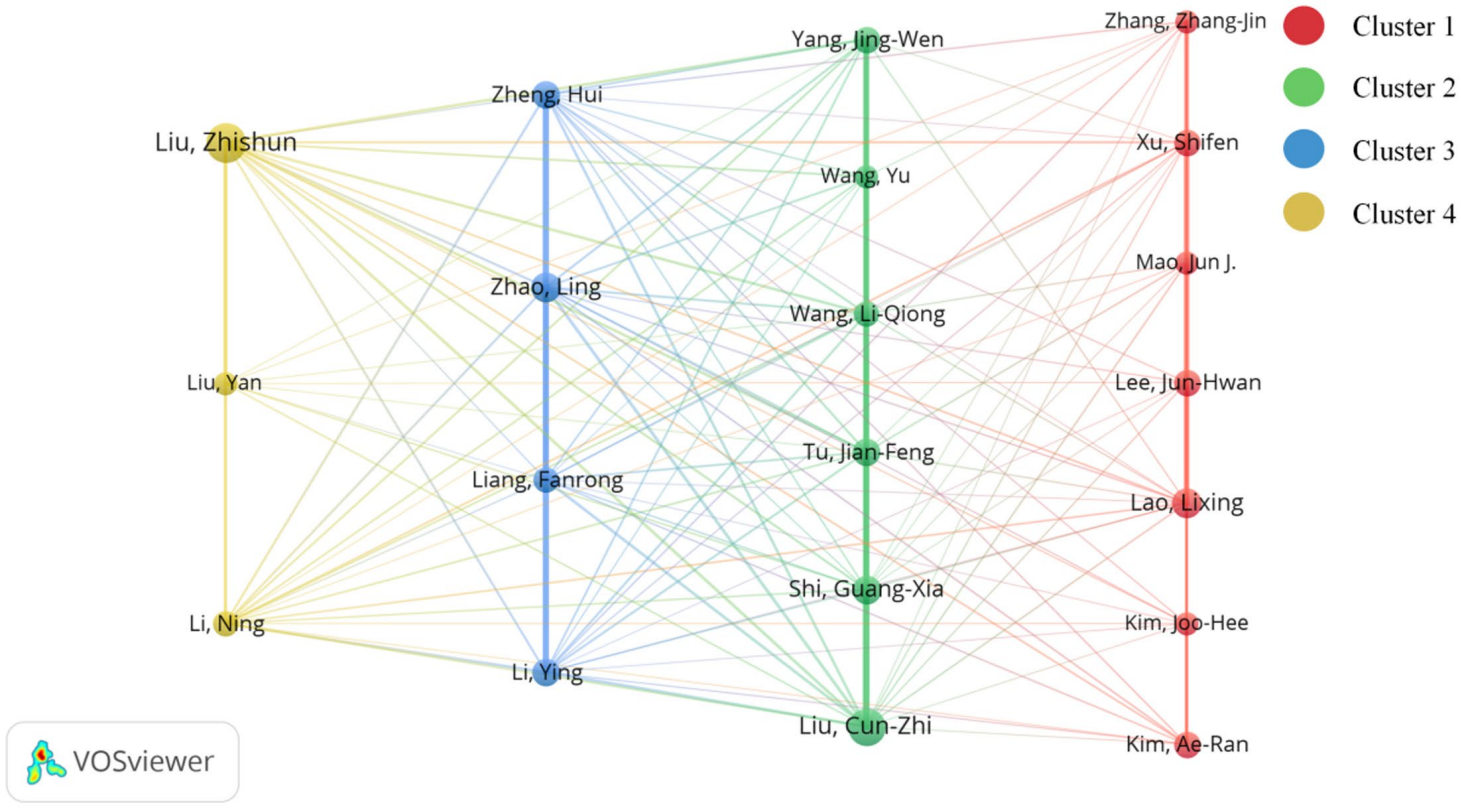
Author co-occurrence map based on citations. To further clarify the co-authorship relationships among core researchers and avoid an overly complex network, a threshold of 20 was selected. Clustering methods: VOSviewer (modularity-based). Node: The number of publications by an author; a larger node indicates more publications. Edge: The link strength between authors; a thicker line indicates a stronger link between authors.

We used VOSviewer to analyze 10,002 authors based on citations, and the co-occurrence analysis revealed that 20 authors had more than 20 publications (Figure 7; Supplementary Table S10). The results show that the author with the most publications is Liu, Zhishun (TP = 53, TC = 788), from the Department of Acupuncture, Guang’an Men’s Hospital, China Academy of Chinese Medical Sciences, China. Liu, Cun-zhi (TP = 48, TC = 831) is the most cited author from Beijing University of Chinese Medicine, China (Table 6).

**Table 6:** The 10 most cited authors in RCTs of acupuncture.

Rank	Authors	Affiliations	TC	TP
1	Liu, Cun-zhi	Beijing University of Chinese Medicine, China	831	48
2	Liang, Fanrong	Chengdu University of Traditional Chinese Medicine, China	824	44
3	Liu, Zhishun	Department of Acupuncture, Guang’an Men’s Hospital, China Academy of Chinese Medical Sciences, China	788	53
4	Li, Ying	Chengdu University of Traditional Chinese Medicine, China	743	28
5	Mao, Jun J.	Integrative Medicine Department, Memorial Sloan Kettering Cancer Center, USA	738	21
6	Zhao, Ling	Chengdu University of Traditional Chinese Medicine, China	721	31
7	Zheng, Hui	Chengdu University of Traditional Chinese Medicine, China	693	27
8	Zhang, Zhang-jin	The University of Hong Kong, Hong Kong, China	588	21
9	Lao, Lixing	The University of Hong Kong, Hong Kong, China	548	32
10	Li, Ning	West China Hospital of Sichuan University, China	534	23

The authors were categorized into four clusters based on affiliations, collaborations, and citations (Figure 7). The 7 authors in Cluster 1 are from Hong Kong, China, the USA, and South Korea. The author with the most publications is Lao, Lixing (TP = 32, TC = 548) from the University of Hong Kong, Hong Kong, China. Mao, Jun J. (TP = 21, TC = 738) is from the Integrative Medicine Department, Memorial Sloan Kettering Cancer Center, USA, with the most citations. All six authors of cluster 2 are from the group of Prof. Liu Cun-zhi (TP = 48, TC = 831) from Beijing University of Chinese Medicine, China. And all four authors in cluster 3 are from Chengdu University of Traditional Chinese Medicine, China, and have strong collaborations among themselves. Liang Fanrong (TP = 44, TC = 824) is an auther with higher research productivity and citation impact. In cluster 4, both Liu, Zhishun (TP = 53, TC = 788) and Liu, Yan (TP = 21, TC = 170) are from the China Academy of Chinese Medical Sciences. They and Li Ning (TP = 23, TC = 534), who is affiliated with West China Hospital of Sichuan University, China, have worked together on the study.

## 4 Discussion

### 4.1 Discussion of annual publications, countries, affiliations, and authors

Since the release of CONSORT 2010 and STRICTA 2010 (15, 25), publications in the last 20 years have ranged from 59 in 2010 to a maximum of 205 in 2022, then plateaued at around 160, with a general upward trend and a tendency toward standardization and normalization of research. Among them, CONSORT 2010 emphasized reporting on key methodological elements of RCTs, such as blind implementation and randomization methods; STRICTA 2010, as an extension of CONSORT, developed 17 entries (six major items) for the complexity of acupuncture interventions, which included, among other things, justification for acupuncture, details of acupuncture, intervention protocols, other details of the intervention, practitioner backgrounds, and interventions in the control group. Their release has greatly improved the quality of reporting, methodological rigor, interpretability of results, and generalizability of acupuncture RCTs (26). With the release of CONSORT 2025 and the medical community’s interest in acupuncture (27), acupuncture RCTs will be further in an active phase in the future.

Following acupuncture’s UNESCO intangible heritage designation in 2010 and enhanced methodological expertise among Chinese researchers (28), China-initiated clinical trials on acupuncture have increased. Analysis of scholarly publications by country revealed China as the top contributor, with the United States and South Korea ranking second and third, respectively. Despite the current multinational interest in acupuncture and the large number of studies conducted to gather evidence-based evidence, international collaboration between countries still needs to be strengthened (29).

From the analysis of organizational affiliation, we identified Beijing University of Chinese Medicine as the organization with the largest number of publications and influence, while the author with the largest number of publications, Liu, Cun-zhi, was also from this organization (Table 6). The cluster analysis in Figure 7 shows that the research team led by Liu Cunzhi in Cluster 2 is largely from the same subject group. They have published many high-level articles in recent years, mainly focusing on functional dyspepsia, postoperative intestinal paralysis, irritable bowel syndrome, osteoarthritis of the knee, and other acupuncture-advantaged diseases (30–33). These efforts have provided rigorous validation and exploration of the therapeutic efficacy, clinical superiority, and dose-effect relationships of acupuncture. In the meantime, their research design is being refined. Intervention modalities comprised manual acupuncture and electroacupuncture (30, 31, 33). Treatment parameters included acupuncture frequency and session duration (33). The control group designs included waiting-list controls and more rigorous sham-acupuncture studies. Among these, sham acupuncture included superficial needling at non-acupoints and non-penetrating placebo devices (e.g., Streitberger needle) (30, 32). Follow-up assessments were emphasized at key time points, typically ranging from 4 to 12 weeks or more after treatment (34). Studying and analyzing the research design of Professor Liu Cunzhi’s team facilitates the development of scientifically rigorous studies, thereby promoting the generation of high-quality evidence.

### 4.2 Discussion of journals and research areas

Analysis of journals reveals a tension between highly centralized publication patterns and low impact. Among the 200 journals we analyzed, the top 10 journals contributed nearly half (47.01%) of the publications. This clearly demonstrates that the research findings of acupuncture RCTs are mainly published in a relatively small number of journal platforms, such as *Trials*, *Medicine*, *Evidence-Based Complementary and Alternative Medicine,* and *Acupuncture in Medicine*, etc. This concentration suggests, on the one hand, that these journals have a positive attitude toward acupuncture RCTs and, on the other hand, that there may be a certain dependence on publication outlets for acupuncture RCT research. However, in-depth analysis of the impact metrics (IF, H-index, TC) reveals that despite their dominance in publication volume, these high-output journals generally exhibit modest publication impact. As presented in Table 3, *Trials*, the highest-output journal (TP = 274), achieves an impact factor of 2.0. Similarly, while *Acupuncture in Medicine* demonstrates the highest H-index (15), its IF is 2.6. Additionally, a discrepancy exists between citation impact (H-index, TC) and publication volume. Although *Trials* published the highest number of articles (*n* = 274), its total citation count (TC = 2090) was comparable to that of *Acupuncture in Medicine* (*n* = 101, TC = 2099). Notably, *Acupuncture in Medicine* achieved a higher H-index (26 vs. 17) with fewer publications. This suggests that the journal may have a higher publication impact. In addition, the H index values (range: 16–26) of high output journals indicate that the sustained publication impact of their articles is relatively limited. Ultimately, a journal’s overall impact is fundamentally determined by the quality of the research it publishes (35).

Our analysis revealed two dominant research domains in contemporary acupuncture RCTs: Integrative Complementary Medicine (TP = 624) and General Internal Medicine (TP = 331). This indicates that acupuncture remains a complementary and alternative therapy within the mainstream medical system (36, 37). Further high-quality evidence is still needed to integrate acupuncture into routine medical practice (38). Concurrently, acupuncture demonstrates strong potential in several key clinical specialties: Neurology (e.g., pain management, stroke rehabilitation), Oncology (e.g., chemotherapy-induced nausea, cancer-related fatigue), Anesthesiology (perioperative analgesia), and Obstetrics Gynecology (dysmenorrhea, pregnancy-related symptoms). This reflects its potential therapeutic effects in neurology, oncology care, anesthetic adjuvant therapy (enhancing efficacy while reducing side effects), and gynecological disease management.

### 4.3 Discussion of keywords and research hotpots

#### 4.3.1 Methodological approaches demonstrate increasing rigor

The prominence of “Randomized Controlled Trial” (439 times), “Protocol” (214 times), and “Double-Blind” (79 times) signifies a critical shift toward standardization. “Protocol” (2020) is the latest keyword to emerge, indicating that the field is progressively focusing on enhancing the reproducibility and rigor of research. This aligns with the SPIRIT and CONSORT extensions for non-pharmacological trials and contributes to trial transparency (27, 39). The emphasis on “sham acupuncture” (69 times) and “multicenter” (52 times) further demonstrates efforts to enhance methodological rigor, and it is essential for generating high-quality evidence to inform clinical guidelines (44 times; burst in 2021).

#### 4.3.2 Expanding the disease spectrum and outcome dimensions in acupuncture research

“Pain” (277 times) ranked as the most frequently occurring condition, with particularly extensive studies on conditions such as knee osteoarthritis, low back pain, and hip disorders (Figure 5A). Recent systematic reviews of acupuncture evidence have demonstrated clinically meaningful efficacy (moderate to large effect sizes) for the following pain conditions: Relief of neck-shoulder pain; Management of myofascial pain syndrome; Reduction of fibromyalgia-related pain; Alleviation of nonspecific low back pain (1). Furthermore, various pain conditions (including but not limited to migraine and cancer-related pain) fall within the established therapeutic scope of acupuncture (40, 41). The mechanisms underlying acupuncture analgesia operate primarily through dual-level modulation: at the peripheral level, involving immunoneuronal interactions, nociceptive ion channels, and endogenous opioid peptide systems (42); within the central nervous system, mediated via TRPV1 signaling, glutamate receptor pathways, glial cell activation, and related neural circuits (43, 44). Despite extensive evidence confirming the efficacy and mechanistic basis of acupuncture analgesia, integrating traditional meridian theory with modern neuroscience in a scientifically rigorous manner remains a critical research challenge.

In addition to analgesia, cluster 1 (Figure 5A) highlights the expansion into neuropsychiatric disorders (“Depression,” “Anxiety,” “Insomnia”), with “Quality of Life” (195 times) as a key outcome. Cluster 3 is also involved in the rehabilitation of neurological disorders such as stroke. Moreover, Cluster 4 emphasizes “Efficacy” (182 times) and “Safety” (57 times) as dual endpoints, which are crucial to improving the credibility of acupuncture research.

#### 4.3.3 Mechanistic and epidemiological integration

The co-occurrence of “Prevalence” (160 times) and “Epidemiology” (43 times) suggests stratification of RCTs by population heterogeneity. In the case of stroke, for example, patients whose complications involve dysphagia, aphasia, and limb dysfunction, effective stratification can demonstrate the utility of acupuncture for different complications (45).

The burst keywords “sleep” and “association” in 2022 highlight the growing interest among researchers in sleep disorders and neurophysiological correlates, such as EEG biomarkers (46, 47). Insomnia is widespread in people with psychiatric or other disorders and has an increased incidence in middle-aged and older adults, perimenopause and menopause, and may lead to complications such as depression and hypertension (48). Several recent studies have incorporated acupuncture as an effective therapy for sleep management (48, 49). Although it has been shown that acupuncture for insomnia may involve neurotransmitters, the hypothalamic–pituitary–adrenal axis, and the intestinal flora, more high-quality trials are needed to further explore its mechanisms (50).

#### 4.3.4 Diverse acupuncture therapies and evidence synthesis

“Electroacupuncture” (409 times) is a form of acupuncture that integrates traditional acupuncture techniques with modern technology, offering the advantage of controllable dosage. Auricular acupuncture, acupressure (Figure 5A, Cluster 1), and other acupuncture methods based on traditional acupuncture theory are also more widely used (51, 52). There are numerous current acupuncture techniques, and how to standardize the indications and explore the mechanism of each technique remains a key issue in the current promotion of acupuncture and its inclusion in guidelines. As shown in Table 5, MA was selected as the intervention modality in nine of the top ten most-cited acupuncture RCTs, highlighting that MA is the primary intervention approach in current research. However, how to promote the standardization of MA protocols, clarify the relationship between dosage and efficacy, and elucidate the specificity of acupoints remains to be further investigated (53).

Meanwhile, in synthesizing evidence from acupuncture research, “Meta-analysis” (108 times) remains the most commonly used method, yet methodological heterogeneity continues to pose a challenge.

### 4.4 Promotion of standardization and quality in acupuncture RCTs

Current research indicates that there remains a need to further enhance the quality of acupuncture randomized controlled trials (26). Identifying key scientific questions and further aligning them with rigorous methodological designs are crucial steps in generating high-quality evidence. Building upon the key content revealed by the co-occurrence map above, our team has focused the discussion on the following three main aspects (potential conditions for acupuncture treatment, key factors influencing acupuncture efficacy, and rigorous methodology) to stimulate reflection among acupuncture researchers.

#### 4.4.1 Identify four types of potential conditions of acupuncture

Based on bibliometric methods such as keyword co-occurrence analysis, burst detection, and document clustering, we have identified four research themes in acupuncture RCTs that exhibit notable research characteristics or development potential. (1) Conditions with conflicting research findings. As shown in Figure 5, knee osteoarthritis (KOA) was a current hot topic in acupuncture RCT research. Although it has now been established that acupuncture (three times a week for eight weeks) is indeed effective for treating KOA (33, 54), the four previously published studies reported conflicting results before this evidence emerged. Studies by Berman (55) and Witt (56) demonstrated that twice-weekly acupuncture sessions could demonstrate the efficacy of acupuncture, whereas research by Scharf (57) and Hinman (58) reported that acupuncture was ineffective (with frequencies of 1.67 and 1.5 sessions per week, respectively). Therefore, in-depth consideration of the key reasons for these conflicting results (the critical scientific questions) helps improve the accuracy of research directions and the reliability of study outcomes. (2) Conditions for which a body of evidence is emerging but has not yet reached a broad consensus. Taking post-stroke dysphagia as an example, this condition carries high risks such as aspiration and aspiration-induced pneumonia, representing the third leading cause of death within the first month after a stroke and significantly reducing patients’ quality of life (59, 60). Although acupuncture therapy has been incorporated into clinical guidelines and is supported by moderate-quality evidence, its recommendation strength remains weak (61). This indicates that the field is in a critical transition phase towards the establishment of clinical norms, and the accumulated research output has laid a solid foundation for subsequent RCTs with higher levels of evidence. (3) Conditions for which modern medicine currently has no superior treatment options. Postherpetic neuralgia is an excruciating pain that primarily affects the elderly population (62). For refractory cases, first-line drugs such as gabapentin often fail to achieve the desired efficacy due to adverse reactions and patient drug tolerance (63). Although existing meta-analyses suggest that acupuncture may offer advantages in alleviating pain and improving treatment efficacy, its application remains limited (64). Meanwhile, research indicates that acupuncture, as a safe adjunctive therapy, may be the optimal treatment approach when combined with antiepileptic drugs for this type of pain (65). Therefore, in such diseases, acupuncture may serve as an important complementary therapy. (4) Conditions where acupuncture can further expand its application. The application of acupuncture in the perioperative period is no longer limited to anesthesia (66). Studies indicate that acupuncture can alleviate adverse psychological states such as tension and anxiety in patients before surgery, reduce the dosage of anesthetic agents and their side effects during surgery, and promote recovery while mitigating postoperative pain during the recovery phase (67). Therefore, during different phases of the perioperative period for various diseases, acupuncture may demonstrate certain therapeutic effects or play a holistic regulatory role, representing a potential direction for future research.

#### 4.4.2 Investigating key factors influencing acupuncture efficacy

With the continuous advancement of acupuncture, the range of available acupuncture interventions has become highly diverse, evolving from ancient stone needles to modern techniques such as manual acupuncture, electroacupuncture, auricular acupuncture, wrist-ankle acupuncture, warming acupuncture, and more (68, 69). For a given condition, selecting different interventions may yield varying therapeutic outcomes. Compared with manual acupuncture, electroacupuncture has superior efficacy in alleviating pain among patients with KOA (70). Meanwhile, each intervention may also have its own unique indications. In the treatment of functional dyspepsia, warm needling is the preferred method for promoting gastric motility (71). It is worth noting that different acupoint prescriptions also influence the efficacy of acupuncture (6). Therefore, it is necessary to further explore the key factors involved in acupuncture during the disease treatment process and investigate its mechanism of action.

Table 5 indicates that in current highly cited research, manual acupuncture remains the predominant choice among acupuncture interventions. As the most widely used acupuncture intervention globally, manual acupuncture is characterized by its ability to adjust stimulation parameters (such as lifting, thrusting, and rotating techniques) in real-time based on patient feedback to maximize therapeutic efficacy (72). However, owing to variations in practitioner experience and potential differences in patient responses (73, 74), achieving standardization and homogenization in manual acupuncture remains a significant challenge currently faced by this technique. The direction of needle insertion, the intensity of stimulation, the depth of insertion, the selection of specific manipulation techniques, the interval between sessions, the duration of needle retention, and the frequency of treatments are all critical components of the acupuncture dose, which is intrinsically linked to its therapeutic efficacy (5, 53). Studies have indicated that 36 acupuncture sessions achieve the optimal therapeutic effect for major depressive disorder, highlighting treatment sessions as a critical factor (75). In KOA, as mentioned previously, acupuncture frequency is the most critical factor influencing therapeutic efficacy. Therefore, clarifying key acupuncture parameters influencing efficacy and exploring their dose-effect relationships, as well as optimal dosage levels, represents a critical scientific questions in current clinical research (76).

#### 4.4.3 Rigorous methodological design facilitates the addressing of key scientific questions in acupuncture

To address well-defined scientific questions, high-quality trials with rigorous designs are crucial for enhancing the credibility of acupuncture research. However, the complex and heterogeneous nature of acupuncture poses significant methodological challenges for RCTs (77, 78). To address these issues, several key aspects require attention.

(1) Adherence to reporting guidelines such as CONSORT and STRICTA is essential for ensuring accurate reporting of randomization, blinding, intervention details, and outcomes. Notably, the CONSORT 2025 statement introduces a new section on patient and public involvement, reflecting methodological advances in clinical practice (79). The efficacy of acupuncture often depends on the patient’s subjective experience and varies according to individual sensitivity to needle stimulation (80, 81). Therefore, integrating this component into trial design may improve the clinical applicability of research findings.
(2) Appropriate control groups should be selected based on the research objectives. When validating the efficacy of acupuncture, sham acupuncture control groups are typically employed (e.g., superficial needling at non-meridian and non-acupoint points, needling at non-acupoint locations, or placebo devices) (82, 83). However, sham acupuncture may still exert certain therapeutic effects (84). Therefore, strict adherence to reporting guidelines for sham acupuncture is necessary to accurately interpret the effects of verum acupuncture (85). When exploring factors influencing the efficacy of acupuncture, it is necessary to compare different levels of that factor, such as treatment frequency (e.g., 3 vs. 5 sessions/week).
(3) Appropriate outcome measures should be selected for specific diseases. For instance, cancer studies may prioritize patient quality of life and mortality rates (86), whereas acute pain-related conditions often focus primarily on immediate symptom relief (87). Meanwhile, “Patient-centeredness” is central to high-quality healthcare, and Patient-Reported Outcomes (PROs) offer a direct means of capturing patients’ self-assessed health status (88). The concept of acupuncture, emphasizing individual experience and holistic regulation, aligns highly with the multidimensional nature of patient-reported outcomes (PROs) (89). Therefore, it is necessary to include PROs within the scope of indicator selection.
(4) Conducting prospective trial registration and data sharing enhances trial transparency and reduces reporting bias. Currently, inconsistencies persist between trial protocols and final publications in acupuncture research (90). Inconsistencies between protocols and publications, along with issues such as low data-sharing rates and “spin” in reporting (26). These findings underscore the need for greater emphasis on trial registration, reporting of results, and data accessibility.

## 5 Limitations

This study has several limitations. First, the data were sourced exclusively from the WOSCC and Scopus, which may exclude relevant studies indexed in other databases (Chinese databases such as CNKI). Second, despite merging synonymous keywords, certain terminology differences may still exist. Third, bibliometric analysis can reveal overall research trends, but it cannot assess the quality of individual studies. Finally, the article included in this study is predominantly in English, which may have resulted in research findings from non-English-speaking regions not being analyzed. Despite these limitations, this study provides a comprehensive analysis and exploration of the hot topics and trends in acupuncture RCTs, offering insights for future research.

## 6 Conclusion

RCTs of acupuncture have been extensively conducted worldwide, with the spectrum of conditions studied continuing to expand (e.g., pain, neurological disorders, mental health conditions). However, to promote the further development of acupuncture, it remains necessary to further clarify key scientific questions in acupuncture research (such as identifying potential conditions for acupuncture and exploring critical factors influencing the efficacy of acupuncture) and address existing methodological challenges. This will facilitate the generation of higher-level evidence for acupuncture and its incorporation into evidence-based guidelines.

## Data Availability

The original contributions presented in the study are included in the article/Supplementary material, further inquiries can be directed to the corresponding author.
