# Supplementary material for: Global research trends and insights in acupuncture randomized controlled trials: a bibliometric analysis
Source: Front Med (Lausanne). 2026 Mar 24;13:1762387. doi: 10.3389/fmed.2026.1762387 (PMC13053237; doi:10.3389/fmed.2026.1762387)
Supplement: Supplementary file 1 [file Supplementary_file_1.docx]

**Supplementary Table S1**

**Comprehensive Search Strategy of WOSCC**

**Database: Web of Science Core Collection (SCI-E, SSCI)**

**Search Date: August 20, 2025**

**Time Span: 2010-01-01 to 2024-12-31**

**Language: English**

| #1 | TS=(acupuncture OR acupuncture therapy OR acupuncture points OR manual acupuncture OR auricular acupuncture OR scalp acupuncture OR electroacupuncture OR acupuncture treatment OR pharmacoacupuncture treatment OR acupotomy) |
| --- | --- |
| #2 | TS=(randomized controlled trial OR RCT OR random allocation) |
| #3 | #1 AND #2 |
| #4 | PY=(2010-2024) |
| #5 | DT=(Article) |
| #6 | LA=(English) |
| Final | #3 AND #4 AND #5 AND #6 |

**Comprehensive Search Strategy of Scopus**

**Database: Scopus**

**Search Date: August 20, 2025**

**Time Span: 2010-01-01 to 2024-12-31**

**Language: English**

| #1 | \| TITLE-ABS-KEY (“acupuncture” OR “acupuncture therapy” OR “acupuncture points” OR “manual acupuncture” OR “auricular acupuncture” OR “scalp acupuncture” OR “electroacupuncture” OR “acupuncture treatment” OR “pharmacoacupuncture treatment” OR “acupotomy”) \| \| --- \| |
| --- | --- | --- |
| #2 | TITLE-ABS-KEY (“randomized controlled trial” OR “RCT” OR “random allocation”) |
| #3 | #1 AND #2 |
| #4 | PUBYEAR > 2009 AND PUBYEAR < 2025 |
| #5 | DOCTYPE (ar) |
| #6 | LANGUAGE (english) |
| Final | #3 AND #4 AND #5 AND #6 |

| **Standardized Name** | **Original Variants Found** |
| --- | --- |
| Liu, Cun-zhi | Liu, Cun-zhi; Liu Cunzhi; Liu CZ; Liu C.; Liu, Cun Zhi |
| Liang, Fanrong | Liang, Fanrong; Liang FR; Liang F.; Liang, Fan Rong |
| Liu, Zhishun | Liu, Zhishun; Liu Zhi-shun; Liu ZS; Liu Z.; Liu, Zhi Shun |
| Li, Ying | Li, Ying; Li Y; Li, Y.; Ying Li |
| Mao, Jun J. | Mao, Jun J.; Mao JJ; Mao, Jun; Jun J Mao |
| Zhao, Ling | Zhao, Ling; Zhao L; Zhao, L.; Ling Zhao |
| Zheng, Hui | Zheng, Hui; Zheng H; Zheng, H.; Hui Zheng |
| Zhang, Zhang-jin | Zhang, Zhang-jin; Zhang ZJ; Zhang Z.; Zhangjin Zhang; Zhang, Zhang Jin |
| Lao, Lixing | Lao, Lixing; Lao LX; Lao L.; Lixing Lao |
| Li, Ning | Li, Ning; Li N; Li, N.; Ning Li |
| Shi, Guang-xia | Shi, Guang-xia; Shi GX; Shi G.; Guangxia Shi; Shi, Guang Xia |
| Xu, Shifen | Xu, Shifen; Xu SF; Xu S.; Shifen Xu; Xu, Shi Fen |
| Yang, Jing-wen | Yang, Jing-wen; Yang JW; Yang J.; Jingwen Yang; Yang, Jing Wen |
| Kim, Ae-Ran | Kim, Ae-Ran; Kim AR; Kim A.; Ae Ran Kim; Kim, Ae Ran |
| Wang, Li-qiong | Wang, Li-qiong; Wang LQ; Wang L.; Liqiong Wang; Wang, Li Qiong |
| Tu, Jian-feng | Tu, Jian-feng; Tu JF; Tu J.; Jianfeng Tu; Tu, Jian Feng |
| Lee, Jun-Hwan | Lee, Jun-Hwan; Lee JH; Lee J.; Jun Hwan Lee; Lee, Jun Hwan |
| Wang, Yu | Wang, Yu; Wang Y; Wang, Y.; Yu Wang |
| Kim, Joo-Hee | Kim, Joo-Hee; Kim JH; Kim J.; Joo Hee Kim; Kim, Joo Hee |
| Liu, Yan | Liu, Yan; Liu Y; Liu, Y.; Yan Liu |

**Supplementary Table S2**

**Standard name list of the top 20 authors**

| **Standardized Name** | **Original Variants Found** |
| --- | --- |
| Beijing University of Chinese Medicine | Beijing Univ Chinese Med; Beijing Univ. Chinese Med.; Beijing Univ Tradit Chinese Med |
| China Academy of Chinese Medical Sciences | China Acad Chinese Med Sci; Chinese Academy of Medical Sciences; Guang'anmen Hospital |
| Shanghai University of Traditional Chinese Medicine | Shanghai Univ Tradit Chinese Med; Shanghai University of TCM |
| Chengdu University of Traditional Chinese Medicine | Chengdu Univ Tradit Chinese Med; Chengdu Univ TCM; Chengdu University of TCM |
| Kyung Hee University | Kyung Hee Univ; Kyung Hee University; Kyunghee University |
| Guangzhou University of Chinese Medicine | Guangzhou Univ Chinese Med; Guangzhou Univ TCM; |
| Capital Medical University | Capital Med Univ; Capital Medical University; |
| Korea Institute of Oriental Medicine | Korea Inst Oriental Med; KIOM; Korean Institute of Oriental Medicine; Oriental Medicine Institute |
| Zhejiang Chinese Medical University | Zhejiang Chinese Med Univ; Zhejiang University of Traditional Chinese Medicine |
| University of Hong Kong | Univ Hong Kong; University of Hong Kong; The University of Hong Kong |
| Tianjin University of Traditional Chinese Medicine | Tianjin Univ Tradit Chinese Med; |
| Sichuan University | Sichuan Univ; Sichuan University; West China Hospital of Sichuan University |
| Nanjing University of Chinese Medicine | Nanjing Univ Chinese Med; |
| China Medical University, Taiwan | China Med Univ Taiwan; China Medical University (Taiwan); |
| Fudan University | Fudan Univ; Fudan University; |
| Daejeon University | Daejeon Univ; Daejeon University; |
| Harvard University | Harvard Univ; Harvard University; Harvard Medical School; Harvard Sch Med; |
| Pusan National University | Pusan Natl Univ; Pusan National University; |
| Chinese University of Hong Kong | Chinese Univ Hong Kong; The Chinese University of Hong Kong |
| Shanghai Jiao Tong University | Shanghai Jiao Tong Univ; Shanghai Jiaotong University |

**Supplementary Table S3**

**Standard name list of the top 20 organizations**

| **Standardized Name** | **Original Variants Found** |
| --- | --- |
| Acupuncture | Acupuncture; Acupuncture therapy; Acupuncture treatment; |
| Randomized controlled trial | Randomized controlled trial; Randomised controlled trial; RCT; Randomized clinical trial; Randomised clinical trial |
| Electroacupuncture | Electro-acupuncture;ElectroAcupuncture; EA; |
| Management | Pain management; Symptom management; Disease management |
| Pain | Pain; Pain intensity; Acute pain |
| Therapy | Therapeutic effect; Intervention; |
| Efficacy | Efficacy; Effectiveness; Clinical efficacy; Therapeutic efficacy; Treatment effect |
| Quality of life | Quality of life; Quality‑Of‑Life; QOL; Health-related quality of life; |
| Prevalence | \| Prevalence; Epidemiology; Incidence; Epidemiological study \| \| --- \| |
| Validation | Validation; Validity; Validation study; Validate |
| Protocol | Protocol; Study protocol; Trial protocol; |
| Depression | Depression; Depressive disorder; Major depressive disorder; |
| Meta-analysis | Meta-analysis; Meta‑analysis; Metaanalysis; Meta analysis; Systematic review and meta-analysis |
| Women | Women; Female; Women's health; Woman |
| Reliability | Reliability; Reproducibility; |
| Stimulation | Needle stimulation; Acupoint stimulation |
| Placebo | Placebo; Placebo effect; Placebo response; |
| Symptoms | Symptoms; Symptom; Clinical symptoms; |
| Complementary | Complementary; Complementary therapy |
| Anxiety | Anxiety; Anxiety disorder; Anxious; Anxiety symptoms |

**Supplementary Table S4**

**Standard name list of the top 20 keywords**

**Supplementary Table S5**

**Standard name list of the top 20 countries**

| **Standardized Name** | **Original Variants Found** |
| --- | --- |
| China | TAIWAN; PEOPLES R CHINA |
| United States | USA; U.S.A.; United States of America; US; U.S. |
| South Korea | South Korea; Korea; Republic of Korea; |
| Germany | Germany; Deutschland; Federal Republic of Germany; |
| Australia | Australia; Commonwealth of Australia; |
| Brazil | Brazil; Brasil |
| United Kingdom | United Kingdom; UK; U.K.; Great Britain; Britain; England; Scotland; Wales; Northern Ireland |
| Iran | Iran; Islamic Republic of Iran; |
| Spain | Spain |
| Canada | Canada |
| Turkey | Turkey; Turkiye; |
| Japan | Japan |
| Switzerland | Switzerland; Swiss Confederation; |
| Sweden | Sweden; Kingdom of Sweden; |
| Denmark | Denmark; Kingdom of Denmark; |
| Norway | Norway; Kingdom of Norway; |
| Israel | Israel |
| Italy | Italy |
| Portugal | Portugal |
| New Zealand | New Zealand |

**Supplementary Table S6**

**Parameter Settings for VOSviewer and CiteSpace**

| **Clustering Software and Methods** | **Parameter Settings** | **Setting details** |
| --- | --- | --- |
| **VOSviewer**  (Modularity-based) | **Scale in visualization** | 1.60 |
|  | **Size variation in labels** | 0.50 |
|  | **Clustering graph** | Circles |
|  | **Lines settings** | size: 0.50  colored lines and straight lines |
| **CiteSpace**  (Spectral Clustering) | **Time slice** | 2010 to 2024 |
|  | **Strength of the link** | Cosine |
|  | **Scope** | Within Slices |
|  | **Selection criteria** | g-index (k=25) |

**Supplementary Table S7**

**Top 20 countries in terms of number of publications**

| Rank | Country | Number of publications | Intermediary centrality | Link Strength |
| --- | --- | --- | --- | --- |
| 1 | China | 1096 | 0.64 | 220 |
| 2 | USA | 263 | 0.30 | 172 |
| 3 | South Korea | 216 | 0.02 | 22 |
| 4 | Germany | 90 | 0.05 | 58 |
| 5 | Australia | 83 | 0.06 | 61 |
| 6 | Brazil | 70 | 0.09 | 15 |
| 7 | United Kingdom | 63 | 0.06 | 32 |
| 8 | Iran | 41 | 0.08 | 25 |
| 9 | Spain | 36 | 0.11 | 13 |
| 10 | Canada | 34 | 0.05 | 29 |
| 11 | Turkey | 27 | 0 | 1 |
| 12 | Japan | 25 | 0 | 111 |
| 13 | Switzerland | 25 | 0 | 28 |
| 14 | Sweden | 24 | 0 | 18 |
| 15 | Denmark | 21 | 0.02 | 17 |
| 16 | Norway | 18 | 0.03 | 17 |
| 17 | Israel | 16 | 0 | 6 |
| 19 | Italy | 16 | 0 | 15 |
| 19 | Portugal | 15 | 0.01 | 10 |
| 20 | New Zealand | 11 | 0 | 10 |

**Supplementary Table S8**

**The 20 Organizations Publishing the Highest Number of Articles about RCTs of Acupuncture**

| Rank | Organization | Records | Citations | Intermediary centrality | Link Strength |
| --- | --- | --- | --- | --- | --- |
| 1 | Beijing University of Chinese Medicine | 166 | 2084 | 0.23 | 226 |
| 2 | China Academy of Chinese Medical Sciences | 121 | 1782 | 0.06 | 166 |
| 3 | Shanghai University of Traditional Chinese Medicine | 121 | 1522 | 0.05 | 113 |
| 4 | Chengdu University of Traditional Chinese Medicine | 116 | 1276 | 0.08 | 105 |
| 5 | Kyung Hee University | 112 | 1329 | 0.04 | 135 |
| 6 | Guangzhou University of Chinese Medicine | 110 | 561 | 0.15 | 71 |
| 7 | Capital Medical University | 96 | 1465 | 0.12 | 140 |
| 8 | Korea Institute of Oriental Medicine Kiom | 76 | 979 | 0.04 | 158 |
| 9 | Zhejiang Chinese Medical University | 62 | 561 | 0.03 | 49 |
| 10 | University of Hong Kong | 60 | 1249 | 0.05 | 124 |
| 11 | Tianjin University of Traditional Chinese Medicine | 55 | 819 | 0.03 | 69 |
| 12 | Sichuan University | 52 | 1110 | 0.03 | 82 |
| 13 | Nanjing University of Chinese Medicine | 47 | 627 | 0.04 | 49 |
| 14 | China Medical University Taiwan | 41 | 452 | 0.09 | 61 |
| 15 | Fudan University | 37 | 890 | 0.01 | 40 |
| 16 | Daejeon University | 32 | 381 | 0 | 73 |
| 17 | Harvard University | 31 | 610 | 0.09 | 23 |
| 18 | Pusan National University | 30 | 625 | 0.02 | 65 |
| 19 | Chinese University of Hong Kong | 29 | 462 | 0.03 | 38 |
| 20 | Shanghai Jiao Tong University | 28 | 444 | 0.01 | 48 |

**Supplementary Table S9**

**The 20 Most Cited Authors in RCTs of acupuncture**

| Rank | Authors | Affiliations | TC | TP | Link Strength |
| --- | --- | --- | --- | --- | --- |
| 1 | Liu, Cun-zhi | Beijing University of Chinese Medicine, China | 831 | 48 | 304 |
| 2 | Liang, Fanrong | Chengdu University of Traditional Chinese Medicine, China | 824 | 44 | 288 |
| 3 | Liu, Zhishun | Department of Acupuncture, Guang’an Men’s Hospital, China Academy of Chinese Medical Sciences, China | 788 | 53 | 113 |
| 4 | Li, Ying | Chengdu University of Traditional Chinese Medicine, China | 743 | 28 | 205 |
| 5 | Mao, Jun J. | Integrative Medicine Department, Memorial Sloan Kettering Cancer Center, USA | 738 | 21 | 32 |
| 6 | Zhao, Ling | Chengdu University of Traditional Chinese Medicine, China | 721 | 31 | 215 |
| 7 | Zheng, Hui | Chengdu University of Traditional Chinese Medicine, China | 693 | 27 | 208 |
| 8 | Zhang, Zhang-jin | The University of Hong Kong, Hong kong, China | 588 | 21 | 76 |
| 9 | Lao, Lixing | The University of Hong Kong, Hong kong, China | 548 | 32 | 109 |
| 10 | Li, Ning | West China Hospital of Sichuan University, China | 534 | 23 | 82 |
| 11 | Shi, Guang-xia | Beijing University of Chinese Medicine, China | 491 | 30 | 182 |
| 12 | Xu, Shifen | Shanghai University of Traditional Chinese Medicine, China | 445 | 26 | 94 |
| 13 | Yang, Jing-wen | Beijing University of Chinese Medicine, China | 389 | 25 | 209 |
| 14 | Kim, Ae-Ran | Korea Institute of Oriental Medicine, Korea | 373 | 26 | 60 |
| 15 | Wang, Li-qiong | Beijing University of Chinese Medicine, China | 361 | 24 | 201 |
| 16 | Tu, Jian-feng | Beijing University of Chinese Medicine, China | 342 | 27 | 214 |
| 17 | Lee, Jun-Hwan | Korea Institute of Oriental Medicine, Korea | 337 | 26 | 38 |
| 18 | Wang, Yu | Beijing University of Chinese Medicine, China | 264 | 21 | 109 |
| 19 | Kim, Joo-Hee | Korea Institute of Oriental Medicine, Korea | 222 | 20 | 43 |
| 20 | Liu, Yan | Beijing University of Chinese Medicine, China | 170 | 21 | 35 |

**Supplementary Table S10**

**The 20 keywords about RCTs of Acupuncture**

| Rank | Keywords | Counts | Link Strength |
| --- | --- | --- | --- |
| 1 | Acupuncture | 1188 | 3668 |
| 2 | Randomized Controlled Trial | 439 | 1435 |
| 3 | Electroacupuncture | 409 | 1290 |
| 4 | Management | 291 | 1164 |
| 5 | Pain | 277 | 1043 |
| 6 | Therapy | 213 | 847 |
| 7 | Efficacy | 182 | 754 |
| 8 | Quality of Life | 156 | 650 |
| 9 | Prevalence | 160 | 614 |
| 10 | Validation | 132 | 520 |
| 11 | Protocol | 125 | 486 |
| 12 | Depression | 102 | 432 |
| 13 | Meta-Analysis | 107 | 427 |
| 14 | Women | 115 | 412 |
| 15 | Reliability | 100 | 395 |
| 16 | Stimulation | 118 | 394 |
| 17 | Placebo | 95 | 369 |
| 18 | Symptoms | 88 | 367 |
| 19 | Complementary | 91 | 365 |
| 20 | Anxiety | 80 | 341 |
